# Embarking on an adventure of early career academic leadership

**DOI:** 10.1530/JME-22-0049

**Published:** 2022-12-07

**Authors:** Tijana Mitić

**Affiliations:** 1 University/British Heart Foundation Centre for Cardiovascular Science, Queen’s Medical Research Institute, University of Edinburgh, Edinburgh, UK

**Keywords:** early career researchers, career progression, research in academia, leadership in crisis, COVID-19 pandemic

## Abstract

Leading a research group as an early career researcher (ECR) in academia presents many challenges. First, it imposes many additional pressures on individuals, causing fear of missing out on a great opportunity that could advance your career. Together, the unsettling nature of short-term or temporary contracts, lack of guidance and the imposter syndrome can trigger a crisis in future leadership. Most leadership positions at universities are held by senior colleagues. ECRs have modest input in decision-making, due to a requirement for specific leadership training and experience with oversight that precedes suitable decision-making. The turbulence of the unprecedented world COVID-19 crisis has been felt disproportionally by many researchers, intensely by those with caring responsibilities. In the current academic climate, navigating either between your postdoctoral or fellowship project, leading others, taking strategic project directions, mentoring or networking may feel like too much. This editorial expresses views on the current state of the matter in academia with suggestions for helpful strategies to employ to meet research endpoints. It also addresses some challenges that new principal investigators and academic leaders may face due to external or institutional change, and provides some tangible advice with action points.

## Invited Author’s profile


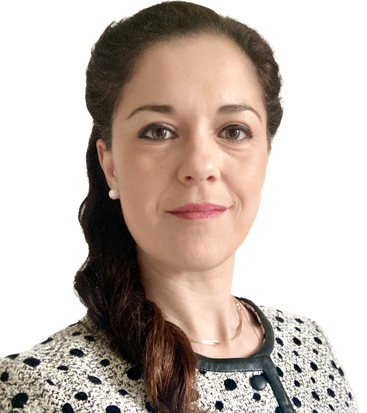


Dr Tijana Mitić obtained her PhD at the Endocrinology Unit of the University of Edinburgh in 2010, followed by a short postdoc immediately afterwards. Following a successful postdoc at the Bristol Heart Institute (2011–2014), Tijana took a break from academia for family reasons. In 2016 Tijana obtained a highly competitive part-time fellowship from the British Heart Foundation, to restart her academic research at the University of Edinburgh, deciphering the epigenetic changes in cardiovascular diseases. In 2019, Tijana was also awarded a Fellowship of the Higher Education Academy for teaching efforts. Her research team investigated the epigenetic changes in hypoxia and role of long non-coding RNAs during vascular injury, at the Centre for Cardiovascular Science at the University of Edinburgh. In 2022 Tijana was awarded a BHF Transitional Fellowship to continue her work on the lncRNA regulation of endothelial cell function. Her team is exploring enhancer-specific lncRNA-actions of relevance for cardiometabolic diseases. Tijana is a *Journal of Molecular Endocrinology* Senior Editor and a 2020 Society for Endocrinology Leadership and Development Awardee.

## Introduction

Good scientific leadership early on in an academic career requires significant discipline. Leading a research project in academia requires a strenuous juggling act, and both male and female colleagues could face similar challenges. However, the global COVID-19 pandemic has brought significant uncertainties, which exacerbated gender differences and challenges associated with scientific leadership (Ahern & Loh 2021). What does sustainable leadership look like if you are feeling trapped and how can you employ clear strategies to meet research endpoints? This article will address the two main challenges which new principal investigators (PIs) and academic leaders are faced with. It provides some tangible advice to mount an active response early on in your leadership role.

## Challenge 1: readiness to lead as an early career academic

The academic journey of an early career researcher (ECR) comprises a wide range of research experiences, a solid training, success with publishing and experience in embracing failure. Securing a renowned personal fellowship and becoming a PI brings a high level of stress and pressure, which strongly depends on the context in which you operate. Although the modern university system recognises many of these pitfalls and guides academics on their career path, not every sector within the university (e.g. HR and promotions, finance, management etc) moves at the same pace forward to offer the support needed by academic leaders. The pre-pandemic climate had already set certain perceptions of how poorly the neoliberal university system was perceived by its academics (Urbina-Garcia 2020). With the COVID-19 pandemic, gender inequality between male and female colleagues was further exposed (Dang & Viet Nguyen 2021). COVID-19 had disproportionally greater impact on female researchers, who felt unsupported to meet research objectives, working remotely, whilst shouldering more of the household and/or childcare duties (Jebsen 2019, Malisch *et al.* 2020).

**Figure 1:**
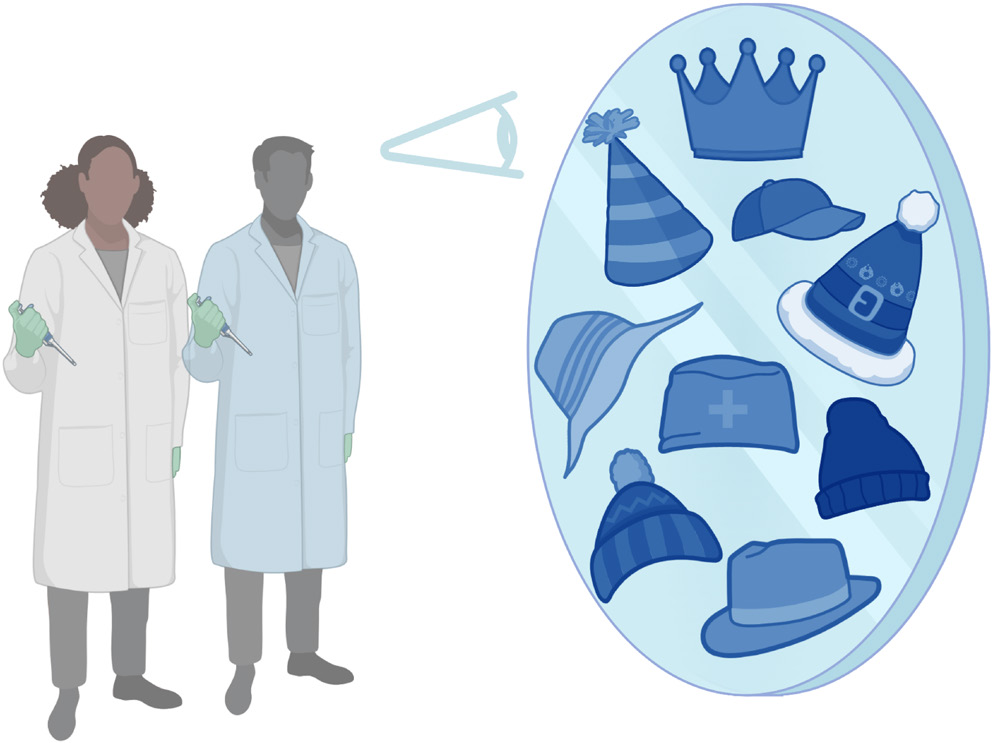
Early career researchers (ECRs) wear multiple hats as leaders from early on in their careers. To perform well and remain successful as leaders, ECRs adopt the roles of team managers, project leaders, specialists in the field, negotiators, research support, data analyst, interviewers, presenters, writers, graphical and text editors as well as entertainers and parents. Image created with Biorender.com

The specific transition from a postdoc or ECR stage, onto a tenure track, is a tough crossroad. Here, many ECR academics could be seen relentlessly adopting multiple performance hats, ranging from being team managers, project leaders, research support, data analyst, negotiators, interviewers, editors, writers etc. (Fig. 1), thus gaining the experience and soft skills to progress in their role.

The pressure on individuals to sustain a successful academic career beyond securing tenure does not ease with seniority. However, low levels of well-being and poor coping strategies have repeatedly been reported amongst ECR groups (Urbina-Garcia 2020). Such effects arise from short-term contracts, parallel clinical engagements, limitations of the length of contract for submitting a project grant to the funder, and also the ongoing impact of the pandemic that could cause a lack of significant funding over time or burnout. All these, combined with managing research at a high pace and a limited lab income, can be very stressful for decision-making. Due to the apparent lack of business and leadership skills amongst academics, there is even greater demand for training to fully realise the potential of this situation (Haage *et al.* 2021). In response to the pandemic and ongoing challenges with delivering academic research, many resilient leaders have abandoned an academic post by securing a biotech or industrial post to prosper in their careers (Gould 2022).

For ECRs, poor job security is a common problem and could limit motivation, due to the lengthy time to transition from a fixed to an open-ended contract. Strategic fulfilment of transition time with unique research ideas and projects that encompass your niche is advised. At this stage, leadership responsibilities and having an open mindset to spread far in your topic could work against you. Equally, their readiness to confront the challenges of solving increasingly complex issues speaks of a dire need for engaging ECRs in a more collaborative leadership and management environment.

## Challenge 2: successful leadership when feeling trapped

At an early stage, ECRs could be expected to propose a solid programme of research with a long-term vision. This could feel unnatural and a tough challenge, especially if you have just secured a personal research fellowship. Having some clear scientific ideas and peer support can be the key. Understanding how you lead your projects will bring you a better sense of control over your research scope and empower you to grow your team. For example, gathering further pilot data on top of recently published manuscripts could look like futureproofing, but it could attest to your clear leadership style to secure a grant application. The grant funding calls are extremely competitive, and many find themselves re-submitting revised proposals and gathering more pilot data. This conundrum could go way beyond the completion of the fellowship due to the uncertain funding landscape and publishing requirements (see Table 1).

**Table 1:** Actions that could ensure publishing successfully

1. Have clear idea of which journals your work fits best
2. Familiarise yourself with the scope of journals in your area
3. Persist with writing cover letters with a unique selling point
4. Establish a connection with editors at meetings and conferences
5. Believe in yourself and your research

In the current research landscape, there are great demands for external funding at any level of seniority, thus creating a mismatch between existing ECRs and new recruits, who often also bring surplus funding (a fellowship or high impact factor paper). Moreover, to meet any chance of an individual securing a tenure position, the system relies on a regular and significant cash injections (project grants, high impact papers, equipment or pump priming funds); a difficult task even for many experienced awardees. Skilful turning of research funds into solid publications is the highest measure of productivity, and that requires time. Perhaps a sensible way to bridge any transition time is to involve service facilities and collaborators in your project. This can help you to temporarily sustain the pressure and successfully meet some research endpoints.

Establishing and running a research group is a process that often reveals who you are. It exposes your natural strengths and abilities to lead others. Hence, authenticity in being yourself around the people you work with, as well as remaining flexible as a leader, is the key to successfully navigate research challenges. The current university system may hold ambiguity and conflicting messaging on requirements for a successful PI. Whilst the work of ECRs is valued solely on their performance and academic outputs, genuinely, there is a great demand for ECRs as efficient academic leaders and managers (see Table 2).

**Table 2:** Desired actions and qualities in ECR leaders

1. Express your ambition: aim for a specific fellowship and progress to it
2. Familiarise yourself with criteria for university promotions
3. Publicise your research and disseminate findings
4. Work on your visibility:
o Develop a team webpage
o Express a clear research vision at relevant meetings
o Engage with industrial partners
o Deposit data in relevant open access research repositories (e.g. Dryad (https://datadryad.org/stash), Zenodo (https://zenodo.org/))
o Track/cite outputs using digital object identifiers (DOIs)
o Regularly update your Linkedin (https://www.linkedin.com/) profile to reach other leaders
o Find your voice (e.g. express it via a blog or on Twitter)

## Available research support for ECRs

It is only through relatedness and self-determination that many ECRs wriggle their way forward in an academic world. With tenure entries raising the bar higher every year (https://www.apa.org/monitor/2019/10/tactics-tenure), researchers are responding by being overworked or displaying a desperate leadership style. Over the past 5 years, many universities have helped early leaders in their research by tackling some of the issues that fall under the remit of the Research Excellence Framework (https://www.ref.ac.uk/). In Scotland, both the University of Edinburgh and the University of Glasgow have accepted responsibility for improving the research culture; this started with an action plan to redesign promotion criteria (Casci & Adams 2020). Many changes were implemented to support researchers in their leadership roles, be it providing a soft skills development programme, funding for teaching qualifications, support for networking, or an early care funding when attending training or a conference.

The biggest recognition and support for research leaders and their careers came in late 2019 in the form of a Researcher Development Concordat (https://researcherdevelopmentconcordat.ac.uk/). The Concordat has set out specific principles on definition of ECRs as both ‘researchers’ and ‘managers of research’, producing action plans which included the highest standards of rigour and research integrity. Many UK universities followed these steps as signatories of Concordat since February 2020. Next, the San Francisco Declaration on Research Assessment (https://sfdora.org) came in, embracing fairer and more reliable approaches for research assessment, although senior management will still be assessed by traditional criteria (Elizabeth 2020). Favoured practical approaches that help us re-imagine a more positive and healthier research culture include the following schemes:

- University mentoring schemes
- EDI (equality, diversity and inclusion) and REC (https://www.advance-he.ac.uk/knowledge-hub/race-equality-charter-review-phase-2) by Wellcome Trust (https://wellcome.org/what-we-do/our-work/research-culture/reimagine-research-culture-festival) and UKRI (https://www.ukri.org/what-we-offer/supporting-healthy-research-and-innovation-culture/equality-diversity-and-inclusion/)
- Vitae research development support (https://www.vitae.ac.uk/policy/concordat) and Inkpath (https://webapp.inkpath.co.uk/)
- Emerging Research Leaders Development Programmes
- Resilient Leaders Development Programme (https://www.resilientleaderselements.com/)

### Role models and active participation

Many ECRs might have been inspired to pursue a career in research upon securing the first large travel grant to showcase final PhD work. Whilst working around successful and inspiring mentors, ECRs may look up to them as role models to follow in their footsteps. However, a typical ECR experience confers that success does not come overnight, nothing is promised, nor does it all come at once. A settlement for a remunerative and highly skilled job (i.e. postdoc) comes with a realisation that it is only a temporary post. Such is the landscape of an early career in academia. However, an aspiring ECR is given a chance to excel quickly in this temporary post, by grasping what the role requests, already planning their next step, and investing additional time/effort to pave the way to a desired research post. Coaches and mentors may help you grow as a leader, and inspire you to perform, but having a short- and long-term vision for your own career is the key. Academics are naturally cautious and discerning, yet honest conversations with a trustworthy mentor/coach can be eye-opening and very healing (see Table 3).

**Table 3:** Motivational encouragement and actions for ECR leaders

1. Avoid getting side-tracked
2. You got here through your own effort
3. Read people and their poker faces
4. Avoid being put down
5. You could be worth more than what you are told
6. Consult a *Mentor*/*Coach* as a sounding board

Considerations before embarking on an academic career:

- What drives you to do independent research?
- What empowers your confidence for research?
- What opportunities are there to fill in the learning gap?
- What does it feel like presenting your science in front of an audience?

### What does a sustainable ECR leadership look like?

**Figure 2:**
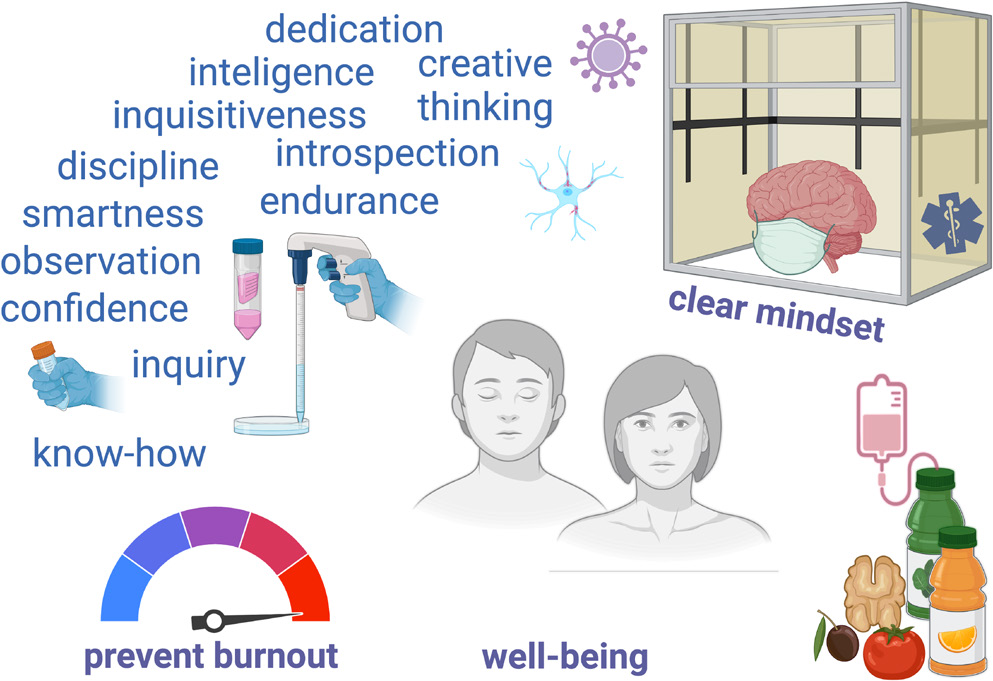
Safeguarding a researchers’ mindset. To prevent any burnout due to named soft and hard skills that researchers have, well-being is to be adopted and a clear mindset created. Image created with Biorender.com. T Mitic is a research fellow at the University of Edinburgh, not a tenured principal investigator, and a research group leader.

Leading a team can feel best when you are in a natural position that provides you with inspiration. One can lead by curiosity, by principle, creativity, inspiring others to act, or demonstrating the commitment to achieve a certain goal. The importance of finding the right environment to thrive in is all, but not even academia will leave you free of management, networking, mentorship, negotiation duties, needing to empower your team, or the necessity for a vision (Haage *et al*. 2021). Striving for lab funding may coincide with re-submitting the revision of your manuscript, and the care and recognition of your team members. Not overreacting to the overload with such tasks, but growing the mindset and behaviour that helps develop a response to each situation is where the change starts; as well as onboarding somebody who can carry part of the managerial burden. The abundance of responsibilities and juggling of multiple leaders’ hats at pre- or early tenure comes with caveats for well-being and the risk of burnout, especially with the extraordinary demands made by the COVID-19 pandemic (Gewin 2021). Some selected values for safeguarding researchers’ mindset are given in Fig. 2 and Table 4.

**Table 4:** Checklist for safeguarding researchers’ mind

Surround yourself with like-minded people
Seek people who share the same values as you
Keep visibility via different media and platforms
Share research ideas openly
Bring answers about an issue you are passionate about

Whilst adapting to leading a research lab, academics are faced with more questions than answers on what actions give success or secure some stability. The current university system offers limited solutions, feeling rather like an adventure embarked on where the details will be figured out later.

## Concluding thoughts

Aspiring to an image of research excellence in a PI, one that fits the requirements of the current academic system, has already had a negative impact on the well-being of researchers (Urbina-Garcia 2020). The motivation for an academic post can be lost over time if individuals are not (yet) tenured, and a wave of big departures from research has already hit academia (Gewin 2022). Universities need to harness more transparent criteria on who they select to progress to ECR, to tenure, or to successive posts. Equally, the current rules of engagement of ECRs with their tenure objectives or endpoints for promotion need to be embedded as a standard practice, to verify a credible intention of any tenure scheme that lasts beyond the next REF (https://www.ref.ac.uk/). Introducing a career pathway for research scientists would be welcomed by many researchers at any university (Casci & Adams 2020). Colleges and universities must act fast to salvage the investment made thus far in a solid training of PhDs and postdocs before contributing further to rampant dropout rates from academia. This is further supported by an apparent paucity of qualified researchers for recruitment to academia (Woolston 2022).

It is apparent that the research culture (https://wellcome.org/reports/what-researchers-think-about-research-culture) within the current university system is overdue for many changes and rethinking (https://www.ukri.org/blog/we-must-reshape-the-system-to-value-and-support-difference/). Just improving your soft skills or your leadership style does not guarantee you success in securing university positions. Moreover, the whole process excludes translational innovators with limited grant or publication records. Hence, retaining more researchers in the academic system, whilst promoting their research efficacy and excellence by allowing time to enhance their outputs, is a way forward. The highly innovative leadership concepts seen in industry and start-ups, corporate or customer-lead roles could be adopted and tailored to academia. This could mount a good response for better engagement of academics and researchers in teaching posts, research spin-off ventures or tenure tracks. In all, there needs to be a coordinated effort between universities, publishers, the government and funders to implement changes across the research community.

Reflecting on the high skill of scientists, it is important to remain aware of the opportunities and success stories of individuals who have secured leadership roles elsewhere in society (Bankston *et al*. 2020). Hence, if the scientific research environment becomes a ground for survival, many other career options remain available. Submitting different applications, going through the interviews with start-ups or research grant submissions hold a huge power. Just going through the process may lead to you feeling empowered or finding a spark, regardless of the outcome or the insecurities of a career transition (away from academia). For those who decide to remain in academia, but require specific development, tailored leadership, a personal coaching programme, or further enhancement of soft skills, that is ok too! It is important to have the courage to explore your choices and find your own path. After all, you deserve to try!

## Conflict of Interest

Tijana Mitić is a Senior Editor of *Journal of Molecular Endocrinology*. She is Society for Endocrinology Leadership and Development awardee for 2020 and a Science Committee member.

## Funding

Tijana Mitić is funded from British Heart Foundationhttp://dx.doi.org/10.13039/501100000274 195CVS/R45927/3102 and the Wellcome Trusthttp://dx.doi.org/10.13039/100010269 Institutional Strategic Funding Award (ISSF3) 195ABR/J22739.
